# Evaluation of Dynamic Respiratory Mechanical Abnormalities During Conventional CPET

**DOI:** 10.3389/fmed.2020.00548

**Published:** 2020-09-10

**Authors:** Kathryn M. Milne, Nicolle J. Domnik, Devin B. Phillips, Matthew D. James, Sandra G. Vincent, J. Alberto Neder, Denis E. O'Donnell

**Affiliations:** ^1^Respiratory Investigation Unit, Division of Respirology, Department of Medicine, Kingston Health Sciences Centre & Queen's University, Kingston, ON, Canada; ^2^Clinician Investigator Program, Department of Medicine, University of British Columbia, Vancouver, BC, Canada; ^3^Laboratory of Clinical Exercise Physiology, Division of Respirology, Department of Medicine, Kingston Health Sciences Centre & Queen's University, Kingston, ON, Canada

**Keywords:** respiratory physiology, dyspnea, respiratory mechanics, inspiratory capacity, cardiopulmonary exercise test

## Abstract

Assessment of the ventilatory response to exercise is important in evaluating mechanisms of dyspnea and exercise intolerance in chronic cardiopulmonary diseases. The characteristic mechanical derangements that occur during exercise in chronic respiratory conditions have previously been determined in seminal studies using esophageal catheter pressure-derived measurements. In this brief review, we examine the emerging role and clinical utility of conventional assessment of dynamic respiratory mechanics during exercise testing. Thus, we provide a physiologic rationale for measuring operating lung volumes, breathing pattern, and flow–volume loops during exercise. We consider standardization of inspiratory capacity-derived measurements and their practical implementation in clinical laboratories. We examine the evidence that this iterative approach allows greater refinement in evaluation of ventilatory limitation during exercise than traditional assessments of breathing reserve. We appraise the available data on the reproducibility and responsiveness of this methodology. In particular, we review inspiratory capacity measurement and derived operating lung volumes during exercise. We demonstrate, using recent published data, how systematic evaluation of dynamic mechanical constraints, together with breathing pattern analysis, can provide valuable insights into the nature and extent of physiological impairment contributing to exercise intolerance in individuals with common chronic obstructive and restrictive respiratory disorders.

## Introduction

Assessment of the ventilatory response to exercise is important in evaluating mechanisms of dyspnea and exercise intolerance in cardiopulmonary diseases (1). The value of the information obtained during cardiopulmonary exercise tests (CPETs) is dependent on the degree to which physiological processes are accurately represented; the quality, reliability, and responsiveness of the measurements; and the interpretation of data to meaningfully impact clinical care. The insights provided by invasive respiratory mechanics using esophageal catheter techniques inform an understanding of respiratory system function during exercise. Although employed in research settings for assessment of respiratory mechanics, during clinical CPETs, esophageal catheter insertion can be cumbersome and time-consuming. Simple, low-cost, non-invasive methods to assess respiratory mechanics in the clinical setting are therefore needed and are the focus of the current review.

Ventilatory limitation is traditionally measured as the ratio of ventilation (V_E_) at peak exercise to measured or estimated maximal voluntary ventilation (V_E_/MVV), with a ratio >85% used to identify ventilation as the cause of reduced exercise capacity (1). Measured MVV during hyperpnea at rest differs from peak exercise V_E_ in respiratory muscle recruitment, operating lung volumes, and breathing pattern (2). Additionally, a high V_E_/MVV provides little information about the specific factors limiting the ventilatory response to exercise in the individual. Patients may perceive intolerable dyspnea during exercise before criteria defining ventilatory limitation are reached. In chronic obstructive pulmonary disease (COPD), 20–50% of patients experience exercise-limiting dyspnea in the setting of sufficient breathing reserve (3, 4). Relying solely on breathing reserve to assess ventilatory response may therefore underestimate physiologic impairment. Measurement of operating lung volumes including end-inspiratory lung volume (EILV), end-expiratory lung volume (EELV), and inspiratory reserve volume (IRV) can be derived from inspiratory capacity (IC) and tidal volume (V_T_) measurement throughout exercise in combination with resting total lung capacity (TLC) (EILV = EELV + V_T_, EELV = TLC – IC, and IRV = IC – V_T_). Exercise flow–volume loops (FVLs) can provide complementary qualitative assessment of airflow limitation (5). Analysis of operating lung volumes, FVLs, and breathing pattern provides insight into mechanical constraints contributing to exercise limitation and dyspnea, avoiding sole reliance on breathing reserve to define ventilatory limitation (5).

Our objective is to provide a brief synopsis of characteristic respiratory mechanical responses to exercise, important assumptions, and limitations involved in measuring operating lung volumes using conventional IC maneuvers, and the rationale for these measurements as they apply to clinical CPET for the frontline clinician. We briefly review recommendations and resources for IC maneuver measurement and available evidence for reliability and reproducibility as well as present a rationale for interpreting operating lung volumes. Finally, we comment on the responsiveness of these dynamic measurements to therapeutic interventions. Other non-invasive methods of assessing respiratory mechanics (e.g., gas dilution techniques and optoelectronic plethysmography) are beyond the scope of this mini-review targeted for clinicians. We direct the interested reader to other recently published reviews on this topic (3, 4).

## Respiratory Mechanics In Health and Disease

### Dynamic Respiratory Mechanics in Health

**Figure 1:**
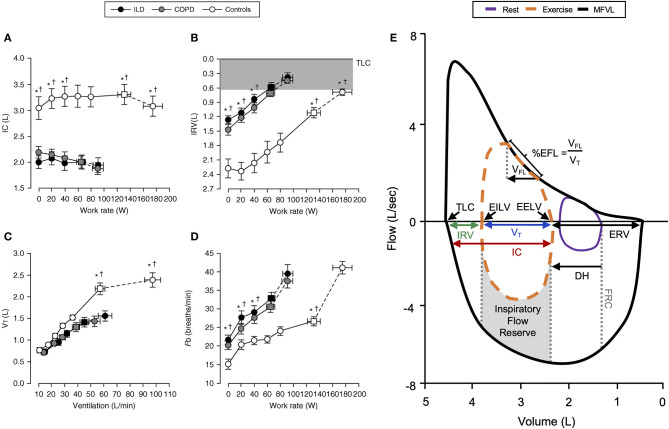
Representative changes in **(A)** inspiratory capacity, **(B)** inspiratory reserve volume, **(C)** tidal volume, and **(D)** breathing frequency in COPD, ILD, and healthy controls during incremental exercise. Note the reduced IC and early critical reduction in IRV with corresponding V_T_ plateau in COPD and ILD subjects. Values are mean ± SEM. *p < 0.05 for ILD vs. control, ^†^*p* < 0.05 for COPD vs. control. Representative flow–volume loop observed in COPD **(E)** demonstrating operating lung volumes, dynamic hyperinflation, and expiratory flow limitation. COPD, chronic obstructive pulmonary disease; DH, dynamic hyperinflation; EELV, end-expiratory lung volume; EFL, expiratory flow limitation; EILV, end-inspiratory lung volume; ERV, expiratory reserve volume; Fb, breathing frequency; FRC, functional residual capacity; IC, inspiratory capacity; ILD, interstitial lung disease; IRV, inspiratory reserve volume; MFVL, resting maximal flow–volume loop; TLC, total lung capacity; V_FL_, volume of tidal breath that is flow limited; V_T_, tidal volume. Reprinted with permission of the American Thoracic Society. Copyright © 2020 American Thoracic Society. All rights reserved Faisal et al. (11). The American Journal of Respiratory and Critical Care Medicine is an official journal of the American Thoracic Society (11). Adapted from Guenette et al. (10).

**Figure 2:**
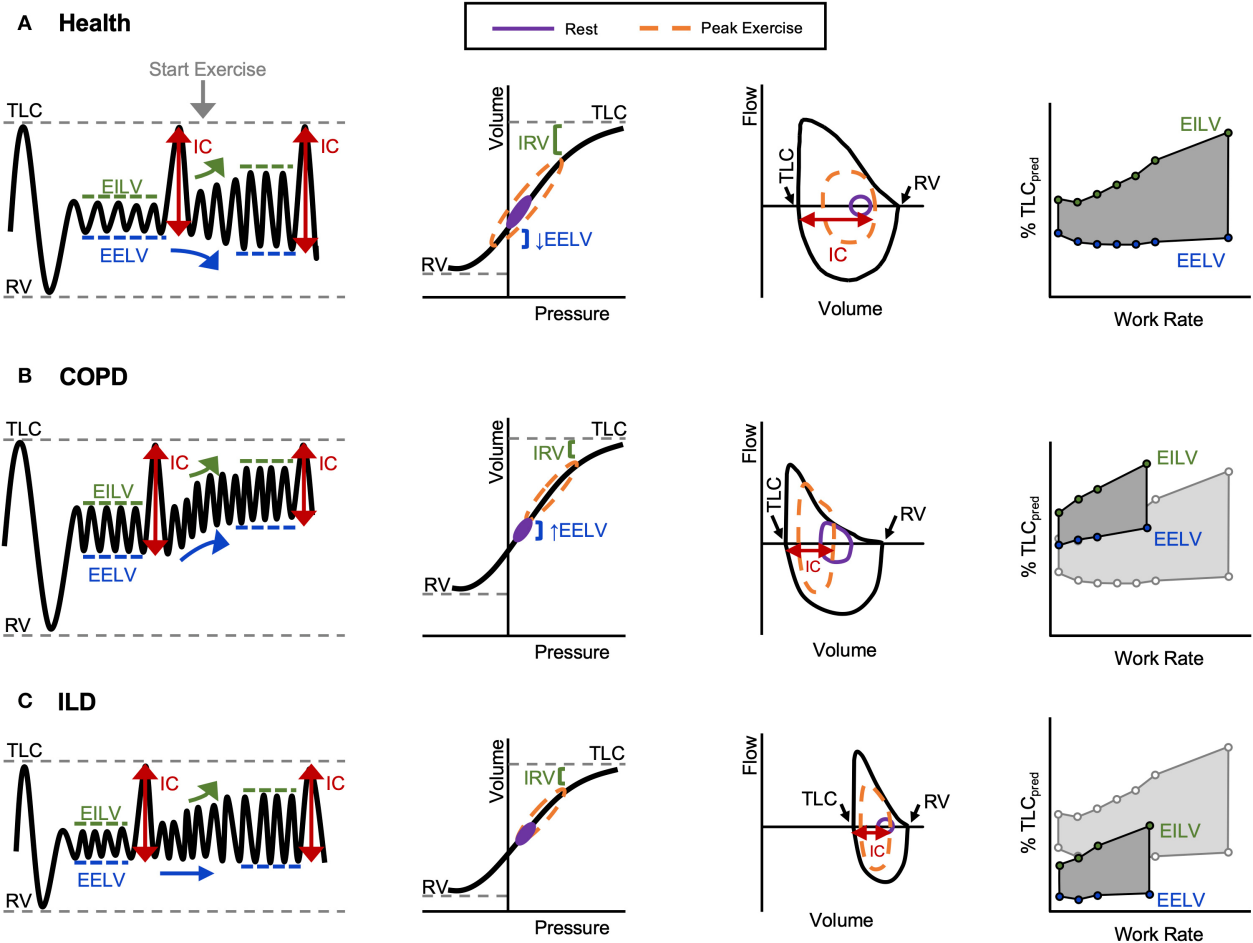
Representative volume–time, pressure–volume, flow–volume, and operating lung volume relationships during exercise observed in **(A)** healthy controls, **(B)** COPD, and **(C)** ILD. COPD, chronic obstructive pulmonary disease; EELV, end-expiratory lung volume; EILV, end-inspiratory lung volume; IC, inspiratory capacity; ILD, interstitial lung disease; IRV, inspiratory reserve volume; RV, residual volume; TLC, total lung capacity. Reprinted from O'Donnell et al. (12). Copyright (2019), with permission from Elsevier (12). Reprinted with permission of the American Thoracic Society. Copyright © 2020 American Thoracic Society. All rights reserved. O'Donnell et al. (13). Proceedings of the American Thoracic Society* is an official journal of the American Thoracic Society. *Now titled Annals of the American Thoracic Society (13).

In health, V_E_ increases in response to the metabolic demands of exercise by increases in V_T_ and breathing frequency (*f*_B_). V_T_ expansion reaches an inflection point at 50–60% of the resting vital capacity (VC), and subsequent rises in V_E_ are secondary to increased *f*_B_ (Figures 1C,D) (6). In young individuals (<35 years old), V_T_ expands with an increase in EILV and decrease in EELV (Figure 2A) (7). In contrast to passive expiration at rest, recruitment of expiratory muscles during exercise leads to a decrease in EELV. This permits V_T_ to expand within the linear compliant portion of the respiratory system pressure–volume curve (Figure 2A). This delays the point during exercise when IRV reaches its lowest value (i.e., EILV is 90–95% of TLC), and the inspiratory muscles must contend with increased elastic mechanical loading (Figure 1B) (8, 9).

FVLs collected during exercise provide a visual representation of V_T_ expansion relative to available capacity. Expiratory flow limitation (EFL) is dependent on the adopted breathing pattern, dynamic EILV and EELV, and maximum FVL (5). EFL can be qualitatively assessed as the percentage of V_T_ over which expiratory airflow is superimposed on or exceeds the maximal flow–volume envelope (Figure 1E) (5). With this approach, establishing an accurate maximal FVL is essential, and without accounting for the influence of thoracic gas compression during forced maneuvers and exercise induced changes in airway caliber overestimation of EFL may result (14). Assessment of maximal FVLs before and after exercise is therefore important. Concavity of the FVL expiratory limb has been associated with dynamic hyperinflation (DH) in severe COPD (15). EFL can be quantitatively assessed using negative expiratory pressure, where application of a standardized negative pressure during expiration and assessment of corresponding changes in expiratory flow are used to determine flow limitation (16–18).

Changes of the respiratory system in healthy aging (>70 years old) have previously been reviewed and include increased lung compliance, decreased chest wall compliance, increased EFL, and elevated ventilatory demand (19). During exercise, EFL occurs at lower V_E_, and both EFL and increased EELV above resting values are more frequently observed at peak exercise in older individuals compared to young adults (20–23). The differences in exertional breathlessness observed in older adults are at least in part related to increased awareness of V_E_ and changes in respiratory mechanical responses during exercise (23–26).

### Dynamic Respiratory Mechanics in Chronic Lung Disease

#### Defining Critical Respiratory Mechanical Constraints

In chronic lung disease, pathology of the lung parenchyma, chest wall, airways, and pulmonary vasculature alter respiratory system compliance, airway resistance, and pulmonary gas exchange, which in variable combination have a deleterious impact on exercise capacity (see reviews in this issue by Devin Phillips, “Measurement and interpretation of ventilatory efficiency during exercise,” and by Denis O'Donnell, “An integrative approach to clinical CPET interpretation”). Increased exertional dyspnea intensity in chronic lung disease is closely related to increased magnitude of inspiratory neural drive (IND) (11) (see review in this issue by Matthew James, “Dyspnea and exercise limitation in COPD: the value of CPET”). Respiratory sensation becomes increasingly unpleasant as neuromechanical dissociation develops, marking the point where increased IND is met with reduced ability of the respiratory system to match increased demand (27). Development of critical respiratory mechanical constraint is defined by the point at which IRV is reduced (within 0.5–1 L of TLC and EILV/TLC > 90–95%), V_T_ expansion has reached an inflection or plateau (occurring at V_T_/IC ~ 70%, identified when V_T_ is plotted against V_E_), and a pronounced increase in dyspnea severity and alteration in its quality (i.e., onset of “unsatisfied inspiration”) occurs (6, 28–30). Importantly, in combination with measures of ventilatory inefficiency, critical mechanical constraints are a more robust predictor of exertional dyspnea and peak VO_2_ compared with breathing reserve (31).

#### Dynamic Respiratory Mechanics in COPD

In COPD, increased lung compliance and EFL due to emphysematous parenchymal destruction and airway remodeling increase the heterogeneity of mechanical time constants for lung emptying. Under the stress of exercise, in the setting of EFL and increased V_E_, there is insufficient time for complete lung emptying, and normal reduction of EELV is impaired (Figures 1E,2B) (32). Progressive increase in EELV during exercise (i.e., DH) undermines the optimal positioning of V_T_ on the pressure–volume curve of the respiratory system (Figure 2B), and dynamic lung compliance decreases while EELV increases in the setting of stable TLC (11, 32, 33). Progressive reduction of IC and IRV during exercise indirectly reflects increased intrinsic elastic mechanical loading of the inspiratory muscles. In flow-limited patients, the IC represents the operating limits for V_T_ expansion during exercise (30, 34, 35). Thus, as IC is reduced, compensatory tachypnea is the only means to increase V_E_ (Figure 1D). Identification of the V_T_ inflection or plateau plotted against V_E_ (Figure 1C) corresponds with the point where IRV reaches a critical minimal (Figure 1B) value in the face of increasing IND. Dynamic respiratory mechanical constraints not only strongly influence the adopted breathing pattern but are also key to the development of exertional dyspnea leading to reduced exercise capacity (30, 36–42).

#### Dynamic Respiratory Mechanics in ILD

In ILD, lung compliance, TLC, and IRV are all reduced, and V_T_ expansion is constrained during exercise reflecting a low IC (11, 43). As a result, critical reduction in IRV manifests early during exercise, and V_T_ is positioned close to the reduced TLC and upper extreme of the contracted pressure–volume curve (Figure 2C). In ILD patients with airway involvement, V_T_ expansion may additionally be restricted due to EFL and an increase in EELV (44, 45). Importantly, in these examples, the classically observed rapid shallow breathing pattern (Figures 1C,D) is a response to the reduced compliance of the respiratory system resulting from restriction of IC and IRV (Figures 1A,B). In both obstructive (COPD) and restrictive (ILD) diseases, “high-end” dynamic mechanics with increased elastic loading, restriction of V_T_ expansion, and relative tachypnea together contribute to functional respiratory muscle weakness, increased work of breathing, IND, and dyspnea (11).

#### Dynamic Respiratory Mechanics in Other Chronic Lung Diseases

Beyond the examples of COPD and ILD, changes in operating lung volumes during exercise have been observed in obesity (46), cystic fibrosis (47), and pulmonary arterial hypertension (PAH) (48). However, unlike healthy, COPD, and ILD populations, the assumptions underpinning IC-derived measurements outlined in the following section have not as yet been robustly investigated in these populations.

## Key Assumptions and Limitations of IC-Derived Operating Lung Volumes

### Validity

IC-derived measurements have been shown to be a valid representation of respiratory mechanics during exercise when performed in conjunction with invasive evaluations using esophageal manometry (29, 49, 50). This has been most thoroughly assessed in COPD patients, and validation of IC-derived techniques in diverse patient populations is needed. IC is determined by the degree of lung hyperinflation and inspiratory muscle strength. Determination of changes in operating lung volumes assumes that changes in IC represent inverse changes in EELV during exercise. Thus, TLC and static inspiratory muscle strength at end-exercise must be similar to values generated at rest for reliable results (7, 33, 49). Stability of TLC during exercise has been demonstrated in healthy, COPD, and ILD populations (33, 45, 46). Furthermore, esophageal pressure (Pes) measured at peak inspired volume plateau (Pes at zero flow following IC) is stable during incremental CPET performed to symptom limitation in COPD (49). When expressed as a percentage of Pes during IC at rest, Pes at symptom limited peak exercise IC exceeds 90% of resting values (49). Additionally, preservation of inspiratory muscle strength assessed using maximal inspiratory pressure (MIP) and sniff Pes pre- and post-exercise demonstrates that respiratory muscle strength can be maintained during exercise (29, 37, 38, 40, 42, 50–53). Taken together, stability of TLC and preservation of Pes-derived assessment of respiratory muscle strength pre- and post-exercise as well as dynamically during exercise IC maneuvers provides evidence that changes in IC reflect changes in operating lung volumes.

### Potential Limitations

Reliable IC-derived measurements additionally assume that maximal volitional effort results in maximal diaphragm activation. The diaphragm has been demonstrated to be maximally activated during voluntary effort in patients with COPD (54). Although maximal voluntary activation of the diaphragm is possible, reproducibility is challenging (55). A recently published study by Luo et al. demonstrated that in some severe COPD patients, IC measurements and associated diaphragm activation (assessed using diaphragmatic electromyography) were submaximal in comparison to supraphysiological experimental stimulation (inhaled 8% CO_2_ gas mixture) (56). Assessing reproducible maximal volitional effort during IC maneuvers can be challenging in the clinical setting, and if patients are not able to perform reproducible IC maneuvers at rest, exercise measurements should not be performed.

Stability of TLC, preservation of maximal voluntary Pes during IC maneuvers, and the voluntary ability to maximally activate the diaphragm support the rationale for using IC maneuvers to measure operating lung volumes. Important clinical scenarios that limit the validity of IC-derived measurements to assess respiratory mechanics include respiratory muscle weakness (failure to successfully reach TLC during IC maneuver can lead to erroneous conclusion of DH), leak during IC maneuver (inability to maintain mouthpiece seal, e.g., bulbar muscle weakness), and inability of the patient to perform reproducible resting IC maneuvers. Additionally, IC-derived measurement of operating lung volumes cannot assess the contribution of chest wall mechanics directly during exercise, and added dead space of mouthpieces may influence breathing patterns. Clinicians should be alert to these situations and consider employing alternative tools for assessment of respiratory mechanics to avoid unreliable operating lung volume measurements.

## Performing High-Quality Reproducible IC Measurements

### Quality Assessment

To obtain reliable and reproducible measurements, IC maneuvers should be performed using a standardized approach. Factors that can interfere with the quality of IC measurements include insufficient instruction, inadequate number of pre-maneuver tidal breaths for assessment of EELV, unstable EELV due to anticipatory changes in breathing patterns, and inadequate effort (10). Quality control considerations, procedures for IC maneuvers, and strategies for ensuring a stable EELV prior to IC measurement are summarized in Table 1 (10, 53). Interested readers are directed to a review by Guenette et al. that describes IC procedures and instructions in detail (10). International guidelines recommend that at least three acceptable resting IC maneuvers be performed and that the mean value of all acceptable resting IC maneuvers be reported (57, 58). IC measurement can be performed during constant work rate (CWR) (59–61) and incremental (62) CPET during both treadmill and cycle exercises (36, 63, 64). During incremental CPET, stepwise increases in work rate as opposed to ramp protocols are preferred so that V_E_ reaches relative stability during each incremental stage when an IC maneuver is performed (10).

**Table 1:** Key steps in IC maneuver performance during CPET.

Prior to IC assessment	Technical considerations	• Use of bidirectional flow-sensing devices for integrated calculation of volume. Measurement of inspiratory and expiratory volumes is important for assessment of EELV and breathing pattern during IC maneuvers (10, 57, 65). • Breath-by-breath cardiopulmonary exercise metabolic system that accounts for thermodynamic drift (5, 65). • The technician conducting the exercise test should be able to view volume–time and/or flow–volume loop tracings preceding and during IC maneuvers.
	Clinical considerations	• Review presence of illness that may impact reliability of IC-derived operating lung volumes during exercise (e.g., respiratory muscle weakness and bulbar muscle weakness). • Consider need for alternative or invasive assessment of respiratory mechanics in patients in whom IC-derived measurements may not be reliable.
Resting IC assessment	Preparation and instructions	1. General description of IC maneuver: *“During the resting period and during each stage of exercise, you will be asked to take a deep breath in until your lungs are completely full. To do this, you will finish your normal breath out then fill up your lungs quickly until you are all the way full. When you can't get any more air in and are completely full, then you can go back to normal breathing”* (10). 2. Demonstration of IC maneuver by technician conducting the exercise test demonstrating normal stable breathing pattern followed by complete inhalation to TLC quickly and without hesitation during IC maneuver. 3. Review instructions for initiation of IC maneuver in order to obtain reproducible measurements at rest. Instructions may be tailored in response to anticipatory changes in breathing pattern by the patient as outlined below (66): • *“At the end of a normal breath out, take a deep breath all the way in until you are completely full”* (10, 53). This instruction may be given when anticipatory changes in breathing pattern are not observed prior to IC maneuvers. • *“At the end of this next breath out, take a deep breath all the way in until you are completely full”* (10, 53). This instruction may be helpful in patients who exhibit anticipatory changes prior to IC maneuvers. • *“Breath all the way in on this breath”* (10). This instruction may be given when anticipatory changes in breathing pattern are not successfully overcome with other sets of instructions, review of the technique, and demonstration. Timing of providing this instruction can be challenging, particularly at high exercise intensities. 4. Repeat resting IC measurement following a minimum of 60 s and only after breathing pattern has returned to pre-maneuver baseline. 5. Verbal encouragement during IC maneuvers to encourage patients to maximally inhale to TLC may be given; however, during research studies, it is particularly important for encouragement to be standardized.
	Quality assessment	• Acceptable IC measurement must not include cough, swallowing, evidence of an obstructed mouthpiece, or mouthpiece leak in the tidal breaths preceding or during the IC maneuver (57). • See *Dynamic IC assessment* below re: EELV. • Although current guidelines do not include reproducibility criteria for resting IC maneuvers (57), values within 10% of the largest acceptable value are frequently used as a threshold for reproducibility (66). • The mean of acceptable values should be reported (57).
Dynamic IC assessment	Preparation and instructions	• Provide instructions for collection of peak exercise IC prior to commencing exercise test: *“During this exercise test the goal is for you to exercise as long as you can until you feel you can't exercise any longer. When you feel you have 10–15 s left, give us a warning wave with your hand so that we can collect the final breathing maneuver”* (10).
	Quality assessment	• EELV assessment prior to IC maneuvers should include a minimum of four tidal breaths (10). • Breathing pattern (depth, frequency, and timing) and EELV should be stable prior to each IC maneuver (10, 65). Anticipatory changes in breathing pattern prior to IC maneuvers can frequently be overcome with adjustment of instructions during preparation at rest, see *Resting IC assessment* above. • EELV during expiration immediately prior to an IC maneuver may frequently overestimate or underestimate EELV, and in this case, the mean EELV for the breaths preceding the IC prompt should be used (65, 66). • Variability in EELV may reflect a mouthpiece leak, and patients should be reminded to maintain a seal on the mouthpiece. • IC measurements following unstable EELV should be discarded. Generally, during dynamic IC measurement, IC maneuvers are not repeated until the next planned interval. • Peak exercise IC during a CPET performed to symptom limitation should be obtained immediately prior to exercise cessation (10).

*CPET, cardiopulmonary exercise test; EELV, end-expiratory lung volume; IC, inspiratory capacity; TLC, total lung capacity*.

### Reproducibility

IC measurements at rest, submaximal, and peak exercise are highly reproducible over time (34, 53, 65, 66). Within-subject coefficient of variation for IC during exercise is 12–20% and has been reported to be higher at end-exercise (34, 65, 66). During CWR CPET performed in a multicenter clinical trial of patients with moderate to severe COPD, IC values at rest, iso-time, and end-exercise are highly repeatable between visits (intraclass correlation *R* ≥ 0.87) (66, 67).

## Interpretation of Operating Lung Volumes

Operating lung volumes can be plotted vs. work rate, oxygen consumption (VO_2_), or V_E_ during exercise (14, 30, 34). Concurrent displays of breathing pattern and FVLs add further refinement to the evaluation of dynamic mechanics. Operating lung volumes should preferably be shown with values derived from healthy age- and sex-matched controls from the same laboratory. When health and disease are compared, expressing values as a percentage of predicted TLC is appropriate, especially when disease alters TLC (10). When reporting individual data, expressing volumes in absolute values or as a percentage of measured TLC may be preferred (10). FVL analysis provides qualitative estimation of EFL and graphic displays of change in operating lung volumes when tidal FVLs are carefully placed on the absolute lung volume axis using serial IC maneuvers and resting TLC (5).

The methodology for describing operating lung volume behavior during exercise is most extensively described in COPD. Change in IC from rest to end-exercise is an accepted assessment of DH (34). An absolute volume threshold to define DH has been debated in the literature. The limitations of an absolute volume definition potentially neglect the importance of interpreting any change in EELV as it relates to development of critical respiratory mechanical constraints, symptoms, and exercise intolerance. Change in IC during exercise should be interpreted in the context of the resting baseline value and critical reduction in IRV during exercise. The advantage of interpreting operating lung volumes and breathing pattern variables together during CPET is the ability to assess the integrated physiologic response to a standardized exercise task. Slopes of IC over time, VO_2_, or V_E_ provide insight into submaximal changes in respiratory mechanics but may not follow a linear relationship (34). In studies comparing effects of bronchodilators with placebo, it is important to additionally assess whether the slope of IC throughout exercise was reduced (i.e., reduced rate of DH) or whether the slope is unchanged but is shifted downward in parallel to placebo, as often is the case. Both a reduced rate of DH and downward shift of EELV and EILV following bronchodilator therapy will delay onset of critical respiratory mechanical constraints, dyspnea, and allow for a longer exercise endurance time.

## Operating Lung Volume Responsiveness To Therapy

In COPD, a low resting IC usually reflects lung hyperinflation, and as a result, V_T_ expansion and increase in V_E_ are limited from the outset of exercise. Resting IC values are correlated with peak VO_2_ (36). Furthermore, IC/TLC is related to mortality, acute exacerbation risk, and development of dyspnea in COPD (30, 68–71). DH is associated with increased mortality (72). Improvement in IC >0.14 L (or 4.5% predicted) exceeds 95% confidence intervals and is associated with significant clinically meaningful improvements in exercise endurance time (36).

Significant improvement in operating lung volumes are highly correlated with reduced exertional dyspnea in COPD following treatment with bronchodilators (34, 59, 60, 73–83). Hyperoxia in both ILD and COPD, by reducing IND and breathing frequency, delays the onset of critical mechanical constraints and extends exercise endurance time (36, 43, 84). In COPD, the effects of bronchodilators and oxygen are additive (85). Exercise training programs in COPD lead to a decrease in IND, V_E_, and breathing frequency, thought to reflect a delay in metabolic acidosis in the subset of patients able to achieve physiologic training effects, in turn delaying onset of ventilatory constraints (86–90). Pulmonary rehabilitation improves exercise capacity in ILD patients, and the ongoing multicenter HOPE-IPF study examines the combined effect of exercise training and oxygen (91–95). During exercise while breathing heliox (21% O_2_ and 79% He) in COPD, a lower gas density of helium leads to decreased airflow resistance, V_E_, and DH (96–98). Bullectomy and lung volume reduction surgery improve static lung elastic recoil, DH, and respiratory muscle function in COPD (99–101). The impact of these interventions and the underlying mechanisms of improvement can be deduced by measuring dynamic respiratory mechanics.

## Conclusions

Operating lung volumes measured throughout exercise provide an assessment of dynamic respiratory mechanics in the clinical setting. IC maneuvers during exercise are simple to perform and, provided sufficient attention is applied, are accurate and reproducible, providing important information about the cause of dyspnea and exercise limitation on an individual basis. Non-invasive measurement of operating lung volumes offers insight into the development of critical respiratory mechanical constraints during exercise, which have been shown to better predict VO_2_ and dyspnea than traditional indices of breathing reserve.

Widespread adoption of conventional IC-derived non-invasive mechanics assessment in clinical CPET awaits development of normative population ranges for operating lung volumes throughout exercise and assessment of reliability in diverse patient populations. Standardized methods for data display and quality control using commercial metabolic carts will facilitate integrating these important physiologic measurements in clinical CPET so as to advance individualized clinical evaluation and management of symptomatic patients.

## Author Contributions

DO'D conceived the idea for the manuscript. KM wrote the first draft of the manuscript. KM, ND, DP, MJ, SV, JN, and DO'D provided critical review and revision of the manuscript. All named authors meet the International Committee of Medical Journal Editors (ICMJE) criteria for authorship for this article.

## Conflict of Interest

DO'D has received research funding via Queen's University from Canadian Institutes of Health Research, Canadian Respiratory Research Network, AstraZeneca, and Boehringer Ingelheim and has served on speaker bureaus, consultation panels, and advisory boards for AstraZeneca and Boehringer Ingelheim. The remaining authors declare that the research was conducted in the absence of any commercial or financial relationships that could be construed as a potential conflict of interest.
